# Osteoblast–Osteoclast Coculture Amplifies Inhibitory Effects of FG‐4592 on Human Osteoclastogenesis and Reduces Bone Resorption

**DOI:** 10.1002/jbm4.10370

**Published:** 2020-05-14

**Authors:** Philippa A Hulley, Ioanna Papadimitriou‐Olivgeri, Helen J Knowles

**Affiliations:** ^1^ Nuffield Department of Orthopaedics Rheumatology & Musculoskeletal Sciences University of Oxford Oxford UK; ^2^ Department of Anatomy Histology & Embryology University of Patras Patras Greece

**Keywords:** 3D MODELS, COCULTURE, OSTEOBLASTS, OSTEOCLASTS, PHD ENZYME INHIBITOR

## Abstract

The link between bone and blood vessels is regulated by hypoxia and the hypoxia‐inducible transcription factor, HIF, which drives both osteogenesis and angiogenesis. The recent clinical approval of PHD enzyme inhibitors, which stabilize HIF protein, introduces the potential for a new clinical strategy to treat osteolytic conditions such as osteoporosis, osteonecrosis, and skeletal fracture and nonunion. However, bone‐resorbing osteoclasts also play a central role in bone remodeling and pathological osteolysis, and HIF promotes osteoclast activation and bone loss in vitro. It is therefore likely that the result of PHD enzyme inhibition in vivo would be mediated by a balance between increased bone formation and increased bone resorption. It is essential that we improve our understanding of the effects of HIF on osteoclast formation and function and consider the potential contribution of inhibitory interactions with other musculoskeletal cells. The PHD enzyme inhibitor FG‐4592 stabilized HIF protein and stimulated osteoclast‐mediated bone resorption, but inhibited differentiation of human CD14+ monocytes into osteoclasts. Formation of osteoclasts in a more physiologically relevant 3D collagen gel did not affect the sensitivity of osteoclastogenesis to FG‐4592, but increased sensitivity to reduced concentrations of RANKL. Coculture with osteoblasts amplified inhibition of osteoclastogenesis by FG‐4592, whether the osteoblasts were proliferating, differentiating, or in the presence of exogenous M‐CSF and RANKL. Osteoblast coculture dampened the ability of high concentrations of FG‐4592 to increase bone resorption. These data provide support for the therapeutic use of PHD enzyme inhibitors to improve bone formation and/or reduce bone loss for the treatment of osteolytic pathologies and indicate that FG‐4592 might act in vivo to inhibit the formation and activity of the osteoclasts that drive osteolysis. © 2020 The Authors. *JBMR Plus* published by Wiley Periodicals, Inc. on behalf of American Society for Bone and Mineral Research.

## Introduction

The skeletal system and the vasculature are strongly linked during bone development and skeletal aging, as well as in bone pathologies such as osteoporosis, osteonecrosis, and skeletal fracture and nonunion.^(^ ^1^ ^)^ Skeletal cells secrete angiogenic factors to stimulate new blood vessel formation. In turn, skeletal blood vessels supply osteoblastic bone‐forming cells with oxygen, nutrients, growth factors, and essential mineralization components such as calcium and phosphate; maintain stem and progenitor cells; and regulate skeletal cell behavior.

The link between blood vessels and bone is regulated by hypoxia and the hypoxia‐inducible transcription factor, HIF.^(^ 2, 3 ^)^ HIF comprises an inducible alpha subunit (HIF‐1α, HIF‐2α) and a constitutively expressed beta subunit. Under standard conditions, HIF‐α is posttranslationally hydroxylated by the prolyl‐4‐hydroxylase enzymes (PHD1–3), targeting it for interaction with the von Hippel–Lindau protein and proteasomal degradation. The PHD enzymes are governed by O_2_ availability, and so exhibit reduced activity under hypoxia. HIF‐α then accumulates, translocates to the nucleus, dimerizes with HIF‐β, and binds the hypoxia‐response element to induce transcription of HIF target genes such as proangiogenic VEGF (reviewed in ref. 4^(^ ^4^ ^)^).

Genetic studies initially defined the HIF‐driven link between osteogenesis and angiogenesis. Mice with osteoblast‐specific overexpression of HIFα caused by deletion of *Vhl* ^(^ ^2^ ^)^ or the *Phd1‐3* enzymes^(^ 5, 6 ^)^ overexpress VEGF and develop dense, heavily vascularized long bones, whereas osteoblast‐specific deletion of *Hif1a* or *Hif2a* produces the reverse phenotype.^(^ 2, 7 ^)^ Similarly, HIF stabilization with PHD enzyme inhibitors increases vascularity and stimulates new bone formation, improving BMD and bone strength in murine models of bone fracture,^(^ 8, 9, 10, 11 ^)^ distraction osteogenesis,^(12^ ^)^ and osteoporosis.^(^ 13, 14 ^)^ The recent approval of novel and specific PHD enzyme inhibitors for clinical use^(^ 15, 16 ^)^ therefore introduces the potential for a new strategy to treat osteolytic diseases.

However, many questions still need to be answered. For instance, some effects of the PHD / HIF pathway on bone are driven not by angiogenesis, but by effects on bone‐resorbing osteoclasts, which play a central role in bone remodeling and pathological osteolysis. Osteoclasts form by fusion of CD14+ monocytic precursors, induced by the cytokines macrophage colony‐stimulating factor (M‐CSF) and RANKL, to produce multinucleated bone‐resorbing cells.^(^ 17, 18 ^)^ By directly comparing HIF knockdown, HIF induction, and PHD enzyme depletion in in vitro cultured murine and human osteoclasts, we showed striking roles for HIF‐1α and PHD2 in driving bone resorption by mature osteoclasts.^(^ 19, 20, 21, 22 ^)^ Osteoclast‐specific inactivation of HIF‐1α antagonizes osteoporotic bone loss in mice, suggesting that HIF‐1α also promotes osteoclast activation and bone loss in vivo.^(^ ^23^ ^)^ Similarly, conditional deletion of *Phd2* in the monocyte/macrophage lineage causes reduced bone mass due to HIF‐mediated production of erythropoietin, which inhibits osteoblast mineralization and induces osteoclastogenesis and bone erosion.^(^ ^24^ ^)^

However, effects of the PHD / HIF pathway on osteoblasts oppose its direct effect on osteoclasts. Mice with an osteoblast‐specific mutation in *Phd2/3* display high bone mass without associated changes in vascularity, instead showing increased mRNA expression and elevated serum concentrations of osteoprotegerin (OPG), an inhibitor of osteoclast formation and activity.^(^ ^5^ ^)^ Reduced numbers of osteoclasts are present in osteoblast‐specific *Phd2/3* ^*−/−*^ mice in vivo, and fewer osteoclasts form in vitro as a result of coculture with *Phd2/3* ^*−/−*^ osteoblasts. This is driven predominantly by direct transcriptional effects of HIF‐2α on *Opg* expression, which does not affect osteoblast proliferation or mineralization.^(^ ^5^ ^)^ We have observed elevated serum concentrations of OPG in *Phd3* ^*−/−*^ mice associated with reduced serum CTXI, indicative of reduced osteoclast activity in vivo.^(^ ^19^ ^)^

It is likely that the result of PHD enzyme inhibition in vivo would be mediated by a balance between increased bone formation and increased bone resorption. Increased trabecular bone mass in mice with an osteoblast‐specific mutation in *Phd1‐3* was described as the result of enhanced osteoclast‐mediated bone resorption exceeded by increased bone formation.^(^ ^6^ ^)^ Any dampening of HIF‐mediated osteoclast activation could encompass a moderate effect of HIF to delay cell fusion during osteoclast differentiation.^(^ ^19^ ^)^

Given the potential of HIF pathway activation as a therapeutic strategy to improve bone formation and/or reduce bone loss, it is essential that we also improve our understanding of the varied effects of HIF on osteoclast formation and function in vivo, especially considering the contribution of inhibitory interactions with other musculoskeletal cell types. Here we describe how a 3D human osteoclast culture or osteoclast–osteoblast coculture can be used to model in vitro some of the nonangiogenic effects of PHD enzyme inhibition in vivo and how it can facilitate detailed evaluation of the molecular mechanisms involved.

## Materials and Methods

### Materials

Reagents were obtained as follows: M‐CSF (R&D Systems, Abingdon, UK), RANKL (Peprotech, London, UK), DMOG (Cayman Chemicals, Ann Arbor, MI, USA), and FG‐4592 (Selleckchem, Houston, TX, USA). Unless stated, other reagents were from Sigma‐Aldrich (Gillingham, UK). Use of leucocyte cones for osteoclast differentiation was approved by the London‐Fulham Research Ethics Committee (11/H0711/7). Leucocyte cones were donated anonymously; no data were available on individual donor age, gender, or health status. Donors were between 50 to 158 kg and 17 to 66 years of age (with some regular donors over 70 years old) and in good health.

### Osteoclast monoculture

CD14+ monocytes were positively selected from the peripheral blood mononuclear cell component of leucocyte cones (NHS Blood and Transplant, Filton, UK) using magnetic CD14+ microbeads (Miltenyi Biotech, Surrey, UK). Monocytes were seeded onto dentin discs or plastic dishes in α‐MEM (without ribonucleosides / deoxyribonucleosides) containing 10% FBS, 2‐mM L‐glutamine, 50‐IU/mL penicillin, and 50‐μg/mL streptomycin sulphate. Osteoclastogenesis was induced by treatment with 25‐ng/mL M‐CSF and 30‐ng/mL RANKL every 3 to 4 days for 9 days. Monocytes were maintained in 25‐ng/mL M‐CSF. Hypoxic exposure was at 2% O_2_, 5% CO_2_, and balance N_2_ in a MiniGalaxy incubator (RS Biotech, Irvine, UK).

### 3D Osteoclast monoculture

There were 1 × 10^6^ CD14+ monocytes pelleted per well of a 24‐well plate, resuspended in 300 μL of 2‐mg/mL collagen type I (Corning, Corning, NY, USA), and allowed to polymerize at 37°C. aMEM and cytokines were applied as for standard osteoclast culture for 9 days. To release cells, gels were digested with 0.2 mg/mL collagenase type I at 37°C for 30 min, washed, and resuspended in α‐MEM prior to reseeding onto plastic or dentin as required. Alternatively, gels were formalin‐fixed, paraffin‐embedded, and sectioned for analysis of internal cells by immunohistochemistry.

## Osteoclast assays

Tartrate‐resistant acid phosphatase (TRAP) staining of formalin‐fixed cells used naphthol AS‐BI phosphate as a substrate, with reaction of the product with fast violet B salt. Photographs were obtained on a Nikon Eclipse TE300 microscope (Nikon, Minato City, Tokyo, Japan) with an Axiocam 105 camera (Carl Zeiss AG, Oberkochen, Germany), and ZEN acquisition software (blue edition; Zeiss). Multinucleated cells containing three or more nuclei were considered osteoclasts. Immunostaining for the vitronectin receptor (VNR) was with anti‐CD51/61 (clone 23C6, 1:400; Bio‐Rad, Oxford, UK) and for cathepsin K with a rabbit polyclonal antibody (3368‐100; BioVision, Milpitas, CA, USA). Measurement of cathepsin K activity as a surrogate for osteoclast number was performed in cells lysed in 1% Triton X‐100 and assayed in an adaptation of previous methods.^(25,26)^ Briefly, 1‐μg lysate was incubated with 100‐μM Z‐Gly‐Pro‐Arg‐7‐(4‐methyl)‐coumarylamide (MCA; Bachem, St Helens, UK), a specific substrate for cathepsin K, in buffer [50‐mM potassium phosphate buffer (pH 6.5), 2.5‐mM DTT, 2.5‐mM EDTA] for 1 hour at 37°C. Generation of fluorescent product was assayed at 380 nm (excitation) and 450 nm (emission).

Resorption tracks on dentin discs were visualized by staining with 0.5% toluidine blue. Dentins were photographed on an Olympus BX40 microscope (Olympus, Shinjuku City, Tokyo, Japan) with ZEN (blue edition) acquisition software; resorption tracks were high‐lighted in Adobe Photoshop (Adobe, San Jose, CA, USA) and the resorbed area was quantified using ImageJ software (NIH, Bethesda, MD, USA; https://imagej.nih.gov/ij/). Alternatively, the release of hydroxyproline was measured as an indication of bone resorption. Sample media was hydrolyzed with HCl and dried, then hydroxyproline concentration was determined by the reaction of chloramine‐T oxidized hydroxyproline with 4‐(dimethylamino)benzaldehyde and the quantification of the colorimetric product at 560 nm.

## Coculture with primary osteoblasts

Primary human osteoblasts were purchased from Sigma‐Aldrich (adult human osteoblasts; 406‐05A) and used at passage 3–7. Donor age was unknown. The same donor vial was used for all experiments. Osteoblasts were titrated to determine the highest viable seeding density to allow continuous culture for the duration of the osteoclast differentiation assay. Osteoblasts were seeded at the determined density in 24‐well plates; 1 × 10^6^ CD14+ monocytes were overlaid 24 hours later. Osteoclastogenesis was induced with three different protocols. (i) Proliferating osteoblasts (seeded at 1 × 10^4^ cells/well) plus 25‐ng/mL M‐CSF and 30‐ng/mL RANKL every 3 to 4 days for 9 days. This enabled direct comparison with 2D osteoclast monoculture; the only difference being the additional presence of osteoblasts (same timescale, same exogenous factors). (ii) Proliferating osteoblasts for 21 days (seeded 500 cells/well), no osteoclastogenic factors. The longer timescale was necessary to enable osteoclast differentiation with the lower concentration of RANKL supplied by the osteoblasts alone. (iii) Differentiating osteoblasts for 21 days (seeded 4 × 10^4^ cells/well), no osteoclastogenic factors. Osteoblast differentiation (mineralization) was induced over the same period by additional treatment with 50‐μg/mL ascorbate and 5‐mM β‐glycerophosphate. Mineralization was measured by calcium quantification.^(^ ^27^ ^)^ Briefly, cells were lysed in 0.1% Triton X‐100, and then mineral was dissolved in 1.0‐N acetic acid. Calcium levels were quantified with a working solution containing o‐cresolphthalein complexone, with absorbance read at 570 nm.

## Western blots

Cells were homogenized in HIF lysis buffer (6.2‐M urea, 10% glycerol, 5‐mM dithiothreitol, 1% sodium dodecyl sulphate, protease inhibitors). Primary antibodies were against HIF‐1α (clone 54, 1:1000; BD Biosciences, Oxford, UK), HIF‐2α (ep190b, Novus Biologicals, Abingdon, UK), GLUT1 (ab14683, 1:2500; Abcam, Cambridge, MA, USA), or β‐tubulin (clone TUB2.1, 1:2500).

## Luciferase assay

Phosphoglycerate kinase is a transcriptional target of HIF‐1α, induced by binding of the transcription factor to its hypoxia‐response element (HRE). As a measure of HIF transcriptional activation, osteoclasts were transfected with a PGK HRE–firefly luciferase plasmid (gifted by Professor AL Harris, Oxford, UK) and a pHRG–TK Renilla luciferase control plasmid (Promega, Southampton, UK) using Lipofectamine 2000 (Invitrogen, Carlsbad, CA, USA ). Luminescence was assayed after 24 to 48 hours using the Dual‐Luciferase Reporter Assay System (Promega), with firefly luciferase normalized to the Renilla transfection control.

## Realtime PCR

RNA was extracted in TRI reagent (Direct‐Zol RNA Miniprep kit; Zymo Research, Irvine, CA, USA), reverse‐transcribed, and qPCR was performed using Fast SYBR Green Master Mix in a Viia7 Real‐Time PCR System (Applied Biosystems, Warrington, UK). Human OPG and RANKL primers were prevalidated Quantitect primers (Qiagen, Manchester, UK). Comparative quantification normalized target gene mRNA to β‐actin (ACTB) mRNA.

## Statistical methods

Results are derived from at least three independent experiments. Data are presented as mean ± SE and were analyzed using GraphPad Prism (GraphPad Software, La Jolla, CA, USA). Statistical analysis comprised one‐way or two‐way ANOVA using Dunnett's or Sidak's multiple comparison as a post hoc test. For experiments with only two conditions, a *t* test was applied. Results were considered significant at *p* < 0.05.

## Results

### FG‐4592 stimulates osteoclast bone resorption, but reduces differentiation

**Figure 1:**
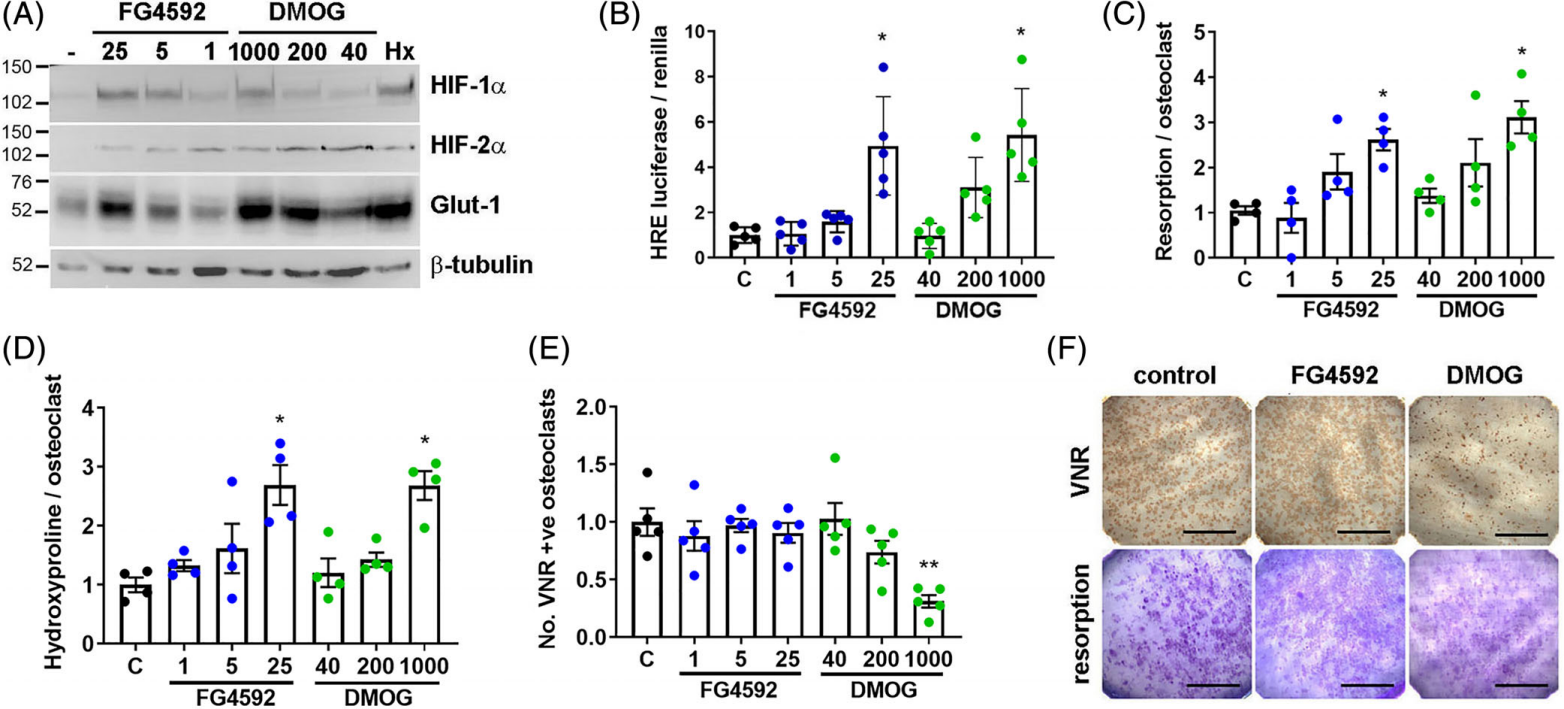
FG‐4592 stimulates bone resorption by mature human osteoclasts. (*A*) Western blot showing stabilization of HIF‐1α and HIF‐2α protein and induction of Glut‐1 and (*B*) HRE‐luciferase activity (*n* = 5) in response to 24‐hour exposure of mature osteoclasts to FG‐4592 (1 to 25 μM), DMOG (40 to1000 μM), or hypoxia (Hx, 2% O_2_). (*C–E*) Quantified effect of 24‐hour exposure of mature human osteoclasts cultured on dentin to FG‐4592 (1 to 25 μM) and DMOG (40 to 1000 μM) with respect to (*C*) the area of dentin resorbed per osteoclast (*n* = 4), (*D*) the amount of hydroxyproline released per osteoclast (*n* = 4), and (*E*) osteoclast survival (number of VNR‐positive osteoclasts present; *n* = 5). (*F*) Representative images of dentin discs after VNR immunohistochemistry or toluidine blue staining of resorption tracks. Scale bar = 1 mm. Each dot represents one independent experiment (ie, one osteoclast donor). VNR = vitronectin receptor. *p < 0.05; **p < 0.01.

Of the classical nonspecific PHD enzyme inhibitors that include dimethyl oxalyl glycine (DMOG), L‐mimosine, desferrioxamine, and CoCl_2_, DMOG causes the greatest increase in osteoclast‐mediated bone resorption, despite some evidence of toxic effects.^(^ 19, 22 ^)^ DMOG is commonly used in vitro at ≤1 mM concentrations to stabilize HIF and activate HIF‐regulated transcription. FG‐4592 (roxadustat, 2‐[(4‐hydroxy‐1‐methyl‐7‐phenoxyisoquinoline‐3‐carbonyl) amino]acetic acid), a more specific PHD enzyme inhibitor that recently obtained clinical approval,^(^ ^16^ ^)^ was titrated to stabilize equivalent quantities of HIF protein in comparison with DMOG (Fig. 1*A*). A 40‐fold lower concentration of FG‐4592 than DMOG induced equivalent HIF transcriptional activation and expression of HIF‐regulated proteins such as Glut‐1 (Fig. 1*A*,*B*). “HIF‐equivalent” doses of FG‐4592 induced an increase in bone resorption by mature osteoclasts comparable to DMOG (Fig. 1*C*,*D*,*F*). Interestingly, although DMOG increases the resorption activity of mature osteoclasts, it also causes a decrease in osteoclast numbers. FG‐4592 did not cause any reduction in the numbers of mature osteoclasts (Fig. 1*E*,*F*).

**Figure 2:**
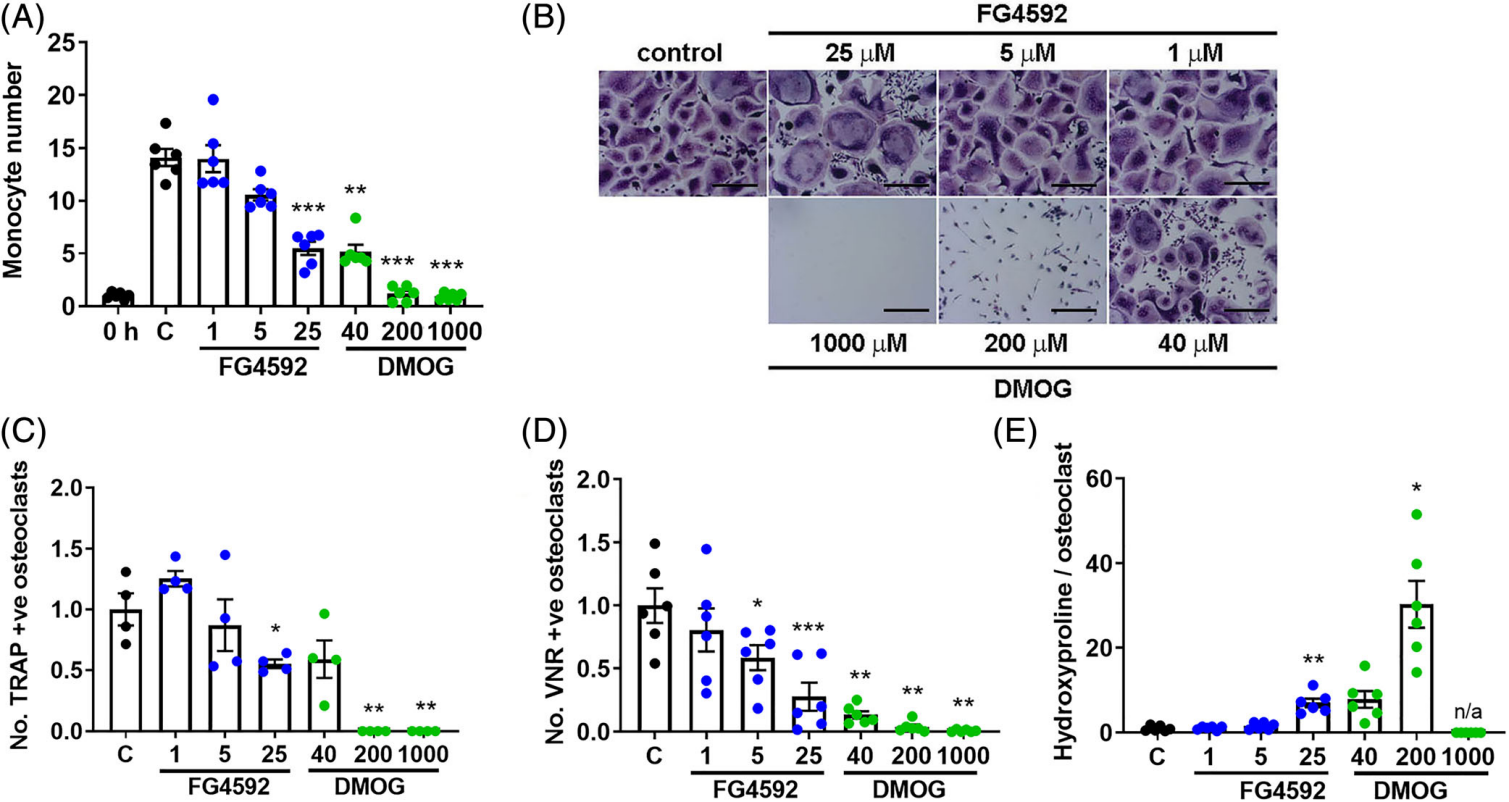
FG‐4592 inhibits osteoclast differentiation. (*A*) Relative number of CD14+ monocytes after 9‐day exposure to FG‐4592 (1 to 25 μM) or DMOG (40 to 1000 μM) (*n* = 6). Significance in comparison to the M‐CSF control (C). (*B*) Representative images of TRAP staining of mature osteoclasts on cell culture plastic. Scale bar = 100 μm. (*C–E*) Quantified effect of 9‐day exposure to FG‐4592 and DMOG during monocyte‐osteoclast differentiation with M‐CSF and RANKL with respect to (*C*) the number of multinucleated TRAP‐positive osteoclasts formed (*n* = 4), (*D*) the number of VNR‐positive osteoclasts formed on dentin (*n* = 6), and (*E*) the amount of hydroxyproline released from dentin per osteoclast from day 7 to day 9 (*n* = 6). Each dot represents one independent experiment (ie, one osteoclast donor). *p < 0.05; **p < 0.01; ***p < 0.001.

The effects of FG‐4592 were next analyzed on osteoclast differentiation. FG‐4592 reduced the rate of proliferation of CD14+ monocyte precursors in the presence of M‐CSF (Fig. 2*A*). FG‐4592 also reduced osteoclast formation induced by M‐CSF and RANKL on both plastic (Fig. 2*B*,*C*) and dentin (Fig. 2*D*) from between 23.3% and 57.9% (25‐uM) and between 58.1% and 84.1% (5‐uM) of control, potentially because of the reduced proliferation of the precursor population. Interestingly, those osteoclasts that did form in the presence of 25‐μM FG‐4592 exhibited a greatly increased capacity for bone resorption (Fig. 2*E*). DMOG caused much greater inhibition of both monocyte proliferation and osteoclast formation, likely because of cumulative toxicity over the 9‐day experimental period, and was therefore not used in further experiments.

### 3D culture increases sensitivity of osteoclastogenesis to RANKL, but not to FG‐4592

**Figure 3:**
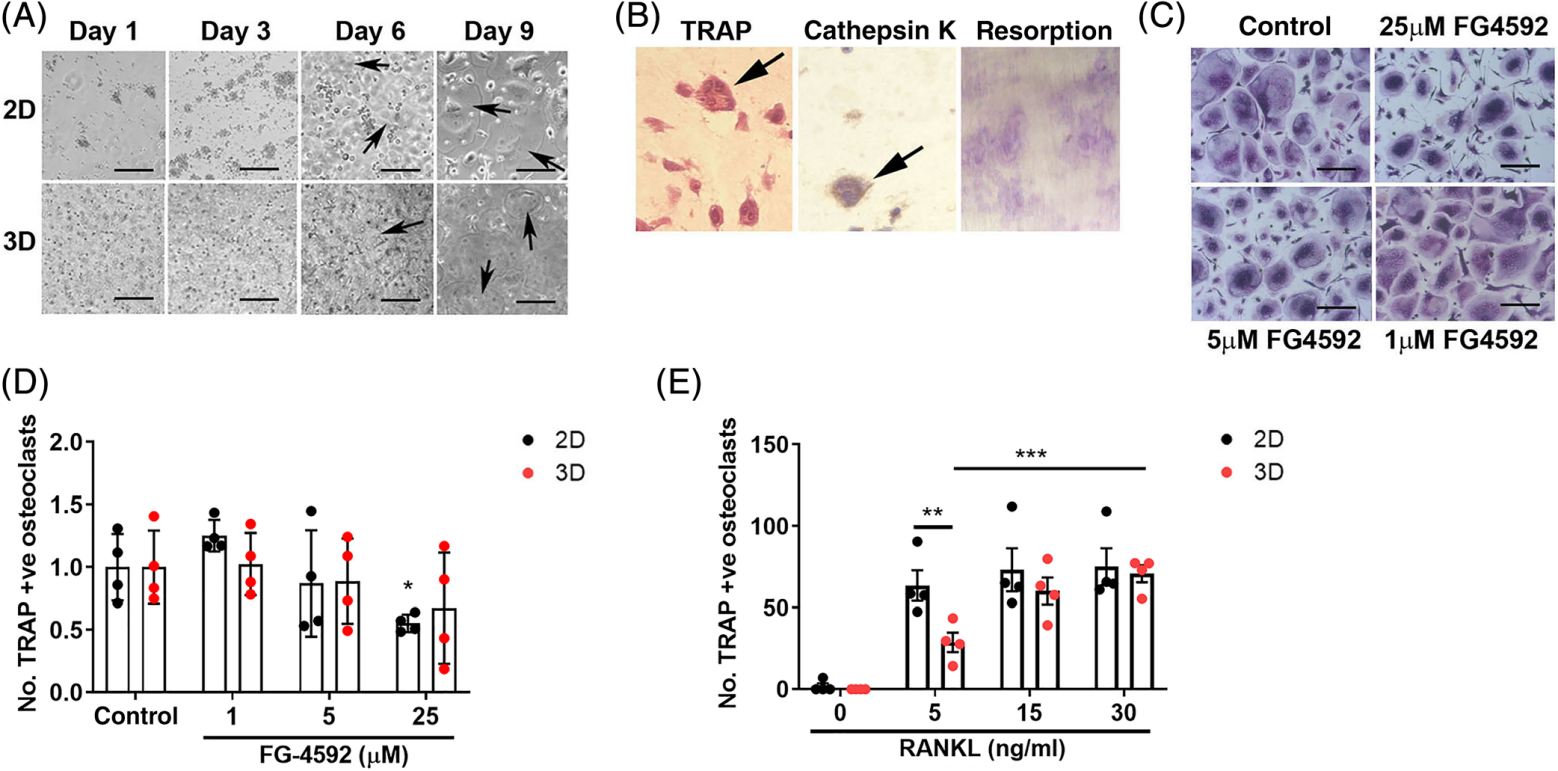
3D Culture alters effects of RANKL on osteoclastogenesis. (*A*) Representative bright‐field image of monocyte–osteoclast differentiation with M‐CSF and RANKL in 2D versus 3D culture. Scale bar = 100 μm. (*B*) Staining of formalin‐fixed paraffin‐embedded 3D gel for TRAP (left panel) and cathepsin K (middle panel). Arrows indicate multinucleated osteoclasts. Right panel: Resorption tracks formed by reseeded osteoclasts on dentin discs. (*C*) TRAP staining of multinucleated osteoclasts differentiated in collagen gels in the presence of FG‐4592 then reseeded in 2D culture. Scale bar = 100 μm. (*D*) Quantification of osteoclasts differentiated in collagen gels with FG‐4592 (*n* = 6) or (*E*) varied concentrations of RANKL (*n* = 4) then reseeded in 2D culture. Comparison with osteoclasts differentiated in 2D culture. Each dot represents one independent experiment (ie, one osteoclast donor). *p < 0.05; **p < 0.01; ***p < 0.001.

We next investigated whether differentiating osteoclasts in a more physiologically relevant 3D culture system might alter effects of PHD enzyme inhibition. By light microscopy, osteoclasts differentiated with M‐CSF and RANKL inside a 2‐mg/mL collagen gel exhibited an equivalent rate of differentiation to those cultured on plastic (Fig. 3*A*). Immunohistochemical analysis of the osteoclasts formed within the gel revealed them to be multinucleated and express the osteoclast markers TRAP and cathepsin K (Fig. 3*B*). These osteoclasts were able to form resorption tracks after release from the gel and reseeding onto dentin discs (Fig. 3*B*). Following gel release, mature osteoclasts were reseeded onto cell culture plastic for quantification of the number of TRAP‐positive osteoclasts formed during 3D differentiation in the presence of FG‐4592 (Fig. 3*C*). No change in the sensitivity of osteoclast formation to FG‐4592 was observed as a result of 3D culture (Fig. 3*D*). However, we did observe an increased sensitivity of osteoclastogenesis to reduced RANKL concentrations in 3D. A 54.7% reduction in osteoclast formation was evident in 3D versus 2D culture at 5‐ng/mL RANKL (*p* < 0.01; Fig. 3*E*). It was not possible to determine direct effects of either treatment on osteoclast activity, as the 6‐day post‐reseeding period necessary to visualize resorption tracks obscured the effects of the 3D culture.

### Coculture with osteoblasts alters effects of FG‐4592 on osteoclastogenesis

**Figure 4:**
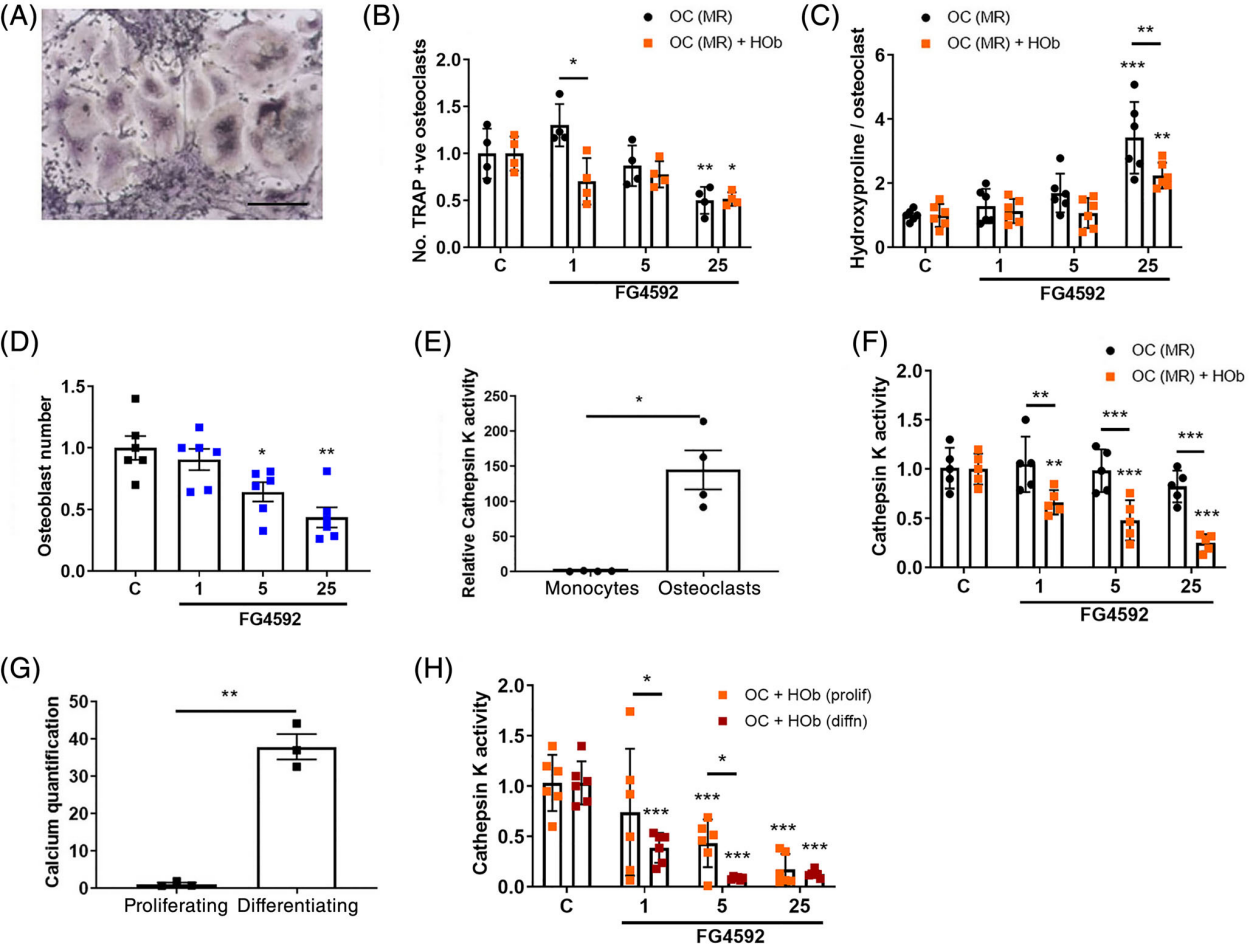
Monocyte–osteoblast coculture amplifies inhibition of osteoclastogenesis by FG‐4592. (*A*) Representative bright‐field image of osteoclast differentiation following monocyte–osteoclast coculture in the presence of M‐CSF and RANKL. Scale bar = 100 μm. (*B*) Relative effect of FG‐4592 (1 to 25 μM) on the number of multinucleated TRAP‐positive osteoclasts formed in osteoclast monoculture versus monocyte‐osteoclast coculture in the presence of M‐CSF and RANKL (*n* = 4). (*C*) Relative effect of FG‐4592 on the amount of hydroxyproline released from dentin (normalized to cathepsin K activity as a reflection of osteoclast numbers) from day 7 to day 9 by osteoclast monocultures versus monocyte‐osteoclast cocultures in the presence of M‐CSF and RANKL (*n* = 6). (*D*) Relative number of osteoblasts after 9‐day exposure to FG‐4592 (1 to 25 μM) (*n* = 6). (*E*) Relative cathepsin K activity measured in monocyte and osteoclast lysates (*n* = 4). (F) Relative effect of FG‐4592 on cathepsin K activity in osteoclast monoculture versus monocyte‐osteoclast coculture in the presence of M‐CSF, RANKL (*n* = 5). (*G*) Relative calcium concentration in cell pellets from differentiated versus proliferating osteoblasts as a measure of mineralization (*n* = 3). (*H*) Relative effect of FG‐4592 on cathepsin K activity in monocyte–osteoclast cocultures in the absence of exogenous M‐CSF and RANKL; proliferating versus differentiating (50 μg/mL ascorbate, 5‐mM β‐glycerophosphate) osteoblast cultures (*n* = 6). Each dot represents one independent experiment (ie, one osteoclast donor). *p < 0.05; **p < 0.01; ***p < 0.001.

We next considered indirect effects of FG‐4592 on osteoclast formation when monocytes were cocultured with osteoblasts. To enable comparison with the direct effects of FG‐4592 on osteoclastogenesis seen in Fig. 2, we first cultured primary human osteoblasts with CD14+ monocytes for 9 days in the presence of exogenous M‐CSF and RANKL. Multinucleated TRAP‐positive osteoclasts formed in the coculture system (Fig. 4*A*), which displayed altered sensitivity to low doses of FG‐4592 (Fig. 4*B*). The osteoclasts that formed in the presence of 25‐μM FG‐4592 also showed dampened resorption activity when cocultured with osteoblasts (Fig. 4*C*). This was not caused by overpopulation of the culture with osteoblasts, which showed a reduced rate of proliferation in the presence of FG‐4592 (Fig. 4*D*).

M‐CSF and RANKL, as well as other pro‐ and anti‐osteoclastogenic factors, are supplied in vivo by cells within the bone microenvironment such as osteoblasts, osteocytes, and T cells. Coculture with osteoblasts is therefore able to stimulate osteoclastogenesis over a 14‐ to 21‐day period in the absence of exogenous M‐CSF or RANKL.^(^ 28, 29 ^)^ It was found that TRAP‐positive osteoclasts could not be visualized directly in many cocultures, but it was not possible to lift the mineralized osteoblast layer without also removing a large proportion of the osteoclasts. Therefore, to enable comparison of results from all coculture systems, lysates were made from osteoclasts or cocultured cells and analyzed for cathepsin K activity, an osteoclast marker enzyme that readily distinguishes mature cells from their monocytic precursors (Fig. 4*E*). Using this method, markedly increased inhibition of osteoclast formation by FG‐4592 was also observed during differentiation with exogenous M‐CSF and RANKL in the presence of osteoblasts (Fig. 4*F*).

Treatment with FG‐4592 had a similar magnitude of effect on osteoclastogenesis during extended coculture with proliferating primary human osteoblasts alone (no exogenous osteoclastogenic cytokines) as with the shorter coculture period in the presence of exogenous cytokines (cathepsin K activity, Fig. 4*H*; compare with Fig. 4*F*). The observed level of inhibition with FG‐4592 was magnified by coculture with differentiating, rather than proliferating, osteoblasts (no exogenous osteoclastogenic cytokines; Fig. 4*G*,*H*).

## Discussion

PHD enzyme inhibitors have now entered clinical use, increasing the feasibility of using HIF pathway activation as a therapeutic strategy to improve bone formation and/or reduce bone loss. However, the complexity of their effects on osteogenic–angiogenic coupling and the potential consequences of actions on the HIF pathway affecting osteoclast:osteoblast interactions are far from clear. Here we have described how the selective PHD enzyme inhibitor FG‐4592 reduces osteoclast differentiation, but enhances osteoclast‐mediated bone resorption in vitro in 2D monoculture. Although 3D monoculture did not alter the effect of FG‐4592 on osteoclast differentiation, 2D coculture with osteoblasts caused amplified inhibition of osteoclastogenesis by FG‐4592 and diminished stimulation of bone resorption.

It is of interest that FG‐4592 caused an increase in bone resorption by mature osteoclasts comparable to DMOG under standard monoculture conditions. DMOG is a 2‐oxoglutarate analogue that acts as a broad‐spectrum inhibitor of 2‐oxoglutarate‐dependent dioxygenases, a family that includes the HIF‐regulating PHD enzymes. DMOG is generally a better mimic of hypoxic transcriptional responses than selective PHD enzyme inhibitors because it includes transcriptional responses mediated by enzymes such as factor‐inhibiting HIF.^(^ ^30^ ^)^ This means that although we titrated the inhibitors to find concentrations that stabilized equivalent amounts of HIF, with comparable induction of Glut‐1 protein and PGK1 HRE luciferase activity, the full panel of downstream target genes induced is likely to diverge. Other groups have observed that FG‐4592 induces expression of HIF‐regulated VEGF and Glut‐1 in vitro over a 5‐ to 100‐μM dose range,^(^ 31, 32, 33, 34 ^)^ equivalent to the concentrations used in this study. FG‐4592 also induces HIF‐mediated expression of erythropoietin and inhibits expression of hepcidin as part of its function to treat anemia in patients with chronic kidney disease.^(^ ^16^ ^)^ The equivalent effect of FG‐4592 and DMOG on osteoclast activity suggests that effects of hypoxia on osteoclast‐mediated bone resorption are driven predominantly by the PHD enzymes, as we have previously reported.^(^ 19, 22 ^)^

Although 3D collagen gel culture did not alter direct effects of FG‐4592 on osteoclastogenesis, it did increase sensitivity to changes in RANKL concentration. We could find no reference in the literature regarding effects of 3D culture on osteoclast biology, although it is known to affect some differentiation and drug‐responses in other cell types. We have previously described enhanced differentiation of primary rat osteoblasts and MC3T3‐E1 cells in 3D versus 2D culture, as well as increased sensitivity to inhibition of proliferation by a protein kinase inhibitor.^(^ ^35^ ^)^ 3D culture alters the sensitivity of prostate cancer cells to antineoplastic drugs,^(^ ^36^ ^)^ and enables spontaneous differentiation of mouse calvarial osteoblasts into osteocytes in the absence of any other factors.^(^ ^37^ ^)^

RANKL is a critical regulator of osteoclast differentiation and activity, whose expression can be regulated in vivo by HIF. HIF‐1α increases RANKL expression in stromal fibroblasts,^(^ ^38^ ^)^ breast cancer cells,^(39^ ^)^ and osteocytic MLO‐Y4 cells.^(^ ^40^ ^)^ HIF‐2α deficiency in mice enhances bone mass, in part by inhibiting osteoclastogenesis. HIF‐2α stimulates RANKL‐induced osteoclastogenesis via regulation of osteoclast Traf6 and also increases RANKL expression in osteoprogenitor cells.^(^ ^41^ ^)^ This could mean that in 3D culture in vivo, the possible effects of FG‐4592 to drive a HIF‐mediated increase in RANKL expression by other bone‐resident cells could have large effects on osteoclast differentiation and activity within the local bone microenvironment.

This might be particularly relevant when considering age‐related bone loss, which could be associated with a reduction in osteoclastogenic RANKL with age. It is assumed that the number and activity of osteoclasts (and osteoblasts) is decreased in the bone tissue of aged individuals; however, a detailed investigation of this hypothesized age difference is lacking. Quantitative gene‐expression analysis and immunohistochemistry of factors related to osteoclastogenesis in human cancellous bone from the distal radius of young (average age 23.2 years) and aged (average age 81.0 years) individuals revealed that RANKL mRNA and protein is significantly higher in young individuals, associated with an increased number of TRAP‐positive osteoclasts.^(^ ^42^ ^)^ However, this does not reflect any difference in the intrinsic ability of old versus young osteoclasts to differentiate ex vivo, when supplied with equal amounts of RANKL and other osteoclastogenic factors. Indeed, the number and size (number of nuclei) of TRAP‐positive osteoclasts generated from human peripheral blood is highly variable, but this is not an age‐dependent effect.^(^ 43, 44, 45 ^)^

As part of our 3D experiments, we developed a method enabling the viable release and reseeding of mature bone‐resorbing osteoclasts from 3D collagen gel culture onto a new bone surface, which they proceeded to digest. Normally osteoclasts grown in vitro lose viability and bone‐resorption activity when detached from the surface. Recent studies have achieved viable release of 2D‐generated osteoclasts using accutase, although there is an indication that murine osteoclasts retain more viability than human osteoclasts following this procedure.^(^ 46, 47 ^)^ The ability to viably move and reseed mature bone‐resorbing osteoclasts in vitro will enable osteoclast differentiation and activity to be studied separately experimentally, a separation that has proven impossible with current osteoclast culture techniques.

The coculture of monocytes with osteoblasts is a well‐known method of generating osteoclasts, utilizing the RANKL supplied by the osteoblastic cells to induce differentiation. Our data—showing that inhibition of osteoclast differentiation by FG‐4592 was greater under coculture with osteoblasts—were similar to that previously described with reagents such as magnesium extract,^(^ ^48^ ^)^ triptolide,^(49^ ^)^ and *Equisetum arvense*.^(^ ^50^ ^)^ It is possible that this is caused by changes in the RANKL:OPG ratio, although our data in this regard were variable and inconclusive (Fig. S1). This could be because expression of both RANKL^(^ 38, 39, 40, 41 ^)^ and OPG^(^ 5, 19 ^)^ can be induced by HIF activation and/or PHD enzyme inhibition. Full transcriptional investigation of this complex experimental system would facilitate identification of all contributing factors.

It would be interesting to combine the methods above to additionally analyze effects of FG‐4592 on osteoclasts (and osteoblasts) in coculture in 3D gels. Unfortunately, we were unable to obtain reproducible differentiation of both osteoclasts and osteoblasts in such a system. Similarly, we obtained highly variable proliferation of undifferentiating osteoblasts in 3D when cocultured with differentiating osteoclasts. Further optimization of this system is necessary to extend the study further in this direction.

In summary, our data provide support for the use of PHD enzyme inhibitors as a potential therapeutic strategy to improve bone formation and/or reduce bone loss during treatment of osteolytic pathologies ranging from cancer and rheumatoid arthritis to osteoporosis and bone fracture. Our human primary cell coculture data indicate that FG‐4592 might act to inhibit the formation and activity of osteoclasts in vivo. In this study, we did not investigate whether it would, in parallel, stimulate angiogenesis and osteogenesis. However, FG‐4592 inhibits the growth of melanoma and lung carcinoma tumors in murine models, in part by normalizing tumor blood vessels,^(^ ^51^ ^)^ and increases vascularity in subcutaneous models of angiogenesis,^(^ ^31^ ^)^ partially via in vivo induction of VEGF.^(^ 31, 33 ^)^ The recent approval of FG‐4592 for clinical use^(^ 15, 16 ^)^ highlights the need to accelerate research into this potential new strategy to treat osteolytic disease.

## Disclosures

PAH was in receipt of a research grant for unrelated work from UCB (R41281CN002) during the conduct of the study. IPO and HJK have nothing to disclose; the authors have no conflict of interest.

## Supporting information


**Figure S1** Changes in the RANKL:OPG mRNA expression ratio in response to FG‐4592. mRNA gene expression ratio of RANKL:OPG in osteoclast: osteoblast cocultures on the final day of differentiation, shown as a fold‐change in cultures treated with 25 μM FG‐4592 versus control. Hob (prolif) = proliferating human osteoblasts, Hob (diffn) = differentiated human osteoblasts, OC = osteoclasts, MR = M‐CSF + RANKL. Osteoclasts express neither RANKL or OPG (therefore the ratio cannot be shown), so all gene expression in the cocultures is derived from osteoblast mRNA.
