# Supplementary material for: Osteoblast–Osteoclast Coculture Amplifies Inhibitory Effects of FG‐4592 on Human Osteoclastogenesis and Reduces Bone Resorption
Source: JBMR Plus. 2020 May 14;4(7):e10370. doi: 10.1002/jbm4.10370 (PMC7340438; doi:10.1002/jbm4.10370)
Supplement: Supplementary file 1 — Figure S1 Changes in the RANKL:OPG mRNA expression ratio in response to FG‐4592. mRNA gene expression ratio of RANKL:OPG in osteoclast: osteoblast cocultures on the final day of differentiation, shown as a fold‐change in cultures treated with 25 μM FG‐4592 versus control. Hob (prolif) = proliferating human osteoblasts, Hob (diffn) = differentiated human osteoblasts, OC = osteoclasts, MR = M‐CSF + RANKL. Osteoclasts express neither RANKL or OPG (therefore the ratio cannot be shown), so all gene expression in the cocultures is derived from osteoblast mRNA. [file JBM4-4-e10370-s001.docx]

**Supplementary Figure 1: Changes in the RANKL:OPG mRNA expression ratio in response to FG-4592.** mRNA gene expression ratio of RANKL:OPG in osteoclast:osteoblast co-cultures on the final day of differentiation, shown as a fold-change in cultures treated with 25μM FG-4592 versus control. Hob(prolif) = proliferating human osteoblasts, Hob(diffn) = differentiated human osteoblasts, OC = osteoclasts, MR = M-CSF+RANKL. Osteoclasts express neither RANKL or OPG (therefore the ratio cannot be shown), so all gene expression in the co-cultures is derived from osteoblast mRNA.
